# Thermophase Seebeck Coefficient in Hybridized Superconductor-Quantum-Dot-Superconductor Josephson Junction Side-Coupled to Majorana Nanowire

**DOI:** 10.3390/nano13172489

**Published:** 2023-09-04

**Authors:** Yumei Gao, Xiaoyan Zhang, Zichuan Yi, Liming Liu, Feng Chi

**Affiliations:** 1 School of Electronic and Information Engineering, UEST of China, Zhongshan Institute, Zhongshan 528400, China; yumeigao@zsc.edu.cn (Y.G.); yizichuan@zsc.edu.cn (Z.Y.); liulmxps@zsc.edu.cn (L.L.); 2 College of Science, North China Institute of Science and Technology, Beijing 101601, China

**Keywords:** thermal phase, seebeck effect, josephson junction, quantum dot, majorana bound states

## Abstract

The dc Josephson current is generated from phase difference between two superconductors separated by a mesoscopic thin film (Josephson junction) without external bias voltage. In the presence of a temperature gradient across the superconductors, a thermal phase is induced under the condition of open circuit. This is very similar to the Seebeck effect in the usual thermoelectric effect, and the thermal phase is thus named as thermophase Seebeck coefficient (TPSC). Here we find obvious enhancement and sign change of the TPSC unique to the Josephson junction composing of two superconductors connected to a semiconductor quantum dot (QD), which is additionally side-coupled to a nanowire hosting Majorana bound states (MBSs), the system denoted by S-MQD-S. These result arise from the newly developed states near the Fermi level of the superconductors due to the QD-MBS hybridization when the dot level is within the superconducting gap. The sign change of the TPSC provides a strong evidence of the existence of MBSs, and is absent if the QD is coupled to regular fermion, such as another QD (system denoted by S-DQD-S). We show that the magnitude and sign of the TPSC are sensitive to the physical quantities including interaction strength between the QD and MBSs, direct overlap between the MBSs, system equilibrium temperature, as well as hopping amplitude between the QD and the superconductors. The obtained results are explained with the help of the current-carrying density of the states (CCDOS), and may be useful in interdisciplinary research areas of Josephson and Majorana physics.

## 1. Introduction

Thermoelectricity is an old research subject about converting thermal bias (temperature difference between different ends of materials) into electrical power, or vice versa [1,2,3]. There are three kinds of closely related thermoelectric effects, of which the most important one is the Seebeck effect. It refers to the generation of an open-circuit voltage ΔV by an applied temperature difference ΔT. The magnitude of the Seebeck effect is denoted by the Seebeck coefficient defined as S=−ΔV/ΔT, whose sign is determined by the kind of the dominant carriers (electrons or holes) and temperature difference. Generally, performances of thermoelectricity in normal metals or superconductors are poor due to the strong particle-hole symmetry [3,4]. In superconductor-based structures, the thermoelectric effect is further weakened by the dissipationless motion of Cooper pairs [3,4]. Therefore, only thermo-phase effects [4,5,6,7,8], which refers to the appearance of phase difference across a Josephson junction to completely cancel the thermocurrent, are usually observed in experiments. To improve the thermoelectric performance by breaking the particle-hole symmetry, particular device designation are required such as suppressing the Josephson coupling in superconducting tunnel junctions [4,5,9] or introducing other materials to form a superconducting hybridized system [10,11].

Diverse superconducting hybridized structures were continuously proposed in the last two decades owing to the rapid development of nano-fabrication technologies [10,12]. One of them is the superconductor-quantum dot (QD)-superconductor (S-QD-S) device [6,8,10,13,14,15,16,17,18]. The energy levels in QD are discrete and controllable with the help of external gate voltage or by changing the dot’s size and host materials [19]. Transportation of quasi-particle and cooper pair in QD-based Josephson junctions has been investigated extensively both theoretically and experimentally in recent years. It was showed that the Josephson current through S-QD-S are well controlled [4,5,6,8,9]. On the other hand, much works were also devoted to phase-coherent thermal transport in superconductor-based circuits [20]. This opens a new subject of phase-coherent thermoelectric devices including heat interferometers [20,21], thermal rectifiers [22], transistors [23], thermometers [24], heat engines [25] as well as refrigerators [26]. Until now, heat interferometers [20,21], thermal diodes [27,28] and a thermal router [29] have been realized experimentally. Superconductor-based thermoelectricity is also promising in thermal logic [30], and provides a means to probe the existence of topological Andreev bound states (ABSs) [31].

In recent years, there are many studies on manipulating and detecting Majorana bound states (MBSs) in systems composing of topological superconductors [32] or hybridized devices with topological properties made from conventional materials, such as semiconductors with strong spin-orbit couplings proximity with normal superconductors [33]. The MBSs are quasiparticles of Majorana fermions that have been realized in various solid-state platforms in recent years [32,33]. They are zero-energy states and neutral in charge, which enable them to be promising in potential applications including fault-tolerant topological quantum computation [34], spintronics [35], and thermoelectricity [36,37,38,39,40,41,42,43]. Just because the above exotic natures of MBSs, the detection of them is still challenging and drawing much attention. One possible signature of the existence of MBSs may be the unusual 4π other than the conventional 2π periodicity of dc Josephson current driven by the phase difference across the junction [43]. A zero-bias conductance anomaly and the corresponding $2e^{2}/h$ quantization of the linear conductance from a normal metallic channel into one mode of the MBSs may be another signature [44].

It was shown that each mode of the MBSs in a topological superconductor carries an extra entropy whose value is independent of temperature [36]. Subsequent work showed that the thermopower between a superconductor and a conducting lead satisfies generally the Mott formula if both normal and Andreev transport processes are considered [37]. This provides an unique way of inferring the temperature of the MBSs. If a Majorana wire is directly connected to external leads with a temperature gradient across them, the magnitudes of thermopower and thermoelectric efficiency depend on configuration of the structure [40]. In systems of a Majorana wire side-coupled to a QD between two normal metal leads (NM-QD-NM) to break the particle-hole symmetry, the sign of the thermopower is reversible by adjusting MBS-MBS hybridization strength [38] or QD-MBSs coupling [42,45]. Under the conditions of weak QD-MBSs coupling and ultra-low temperature regime, both the thermopower and thermoelectric efficiency can be significantly enhanced [42]. The sign change of the thermopower and tunable thermoelectric conductance by parameters related to the MBSs serves as a strong evidence of their existence.

**Figure 1:**
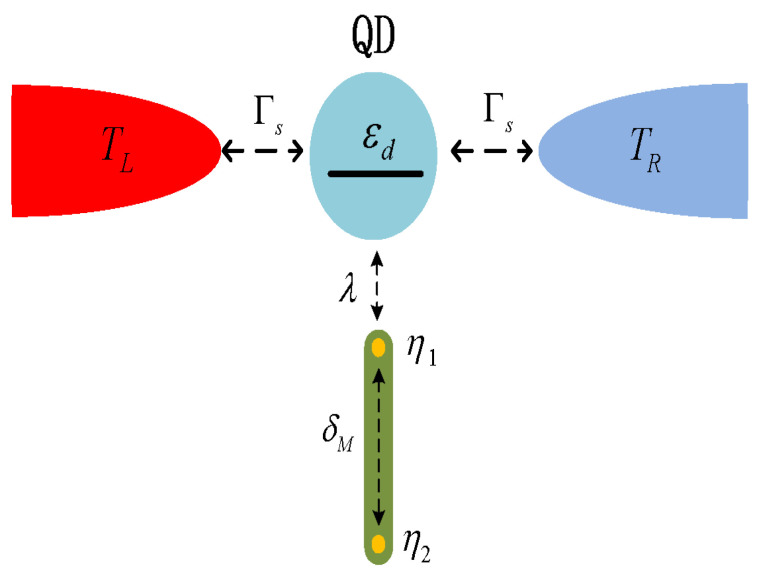
(Color online) Schematic plot of the S-MQD-S system composing of a single-level QD that is connected with strength $\Gamma_{s}$ to the left and right superconductor leads held at $T_{L}$ and $T_{R}$, and further coupled to a nanowire with MBSs at its ends with strength λ. The MBSs denoted by $\eta_{1/2}$ hybridize to each other with amplitude of $\delta_{M}$.

In view of the successful applications of thermoelectric effect for detecting MBSs in NM-QD-NM, here we consider the same issue in S-MQD-S system as shown in Figure 1. In analogous to the physical quantity of thermopower in NM-QD-NM that measures the induced bias voltage by temperature difference, the thermophase Seebeck coefficient (TPSC) in S-MQD-S is studied with particular attention paid on its sign change. Previous work showed that the magnitude of the TPSC in S-MQD-S depends on the dot level, coupling between the QD and superconductor leads, and system temperature [6,8]. Moreover, the sign of the TPSC is sensitive to the temperature [6]. In system of S-DQD-S, the magnitude of TPSC is adjustable with the help of the energy level of the side-coupled dot [8]. Whereas in the present S-MQD-S, our numerical results show that the sign of TPSC can be effectively reversed by variation of the direct MBS-MBS hybridization, QD-superconductor coupling in addition to the system temperature. We emphasize that this sign change of the TPSC can hardly appear in system of S-DQD-S [8], and then might potentially be associated with the presence of MBSs.

## 2. Model and Method

We consider the S-MQD-S structure shown in Figure 1, i.e., a spinless single-level QD is sandwiched between the left and right Bardeen-Cooper-Schrieffer (BCS) superconductors and side-coupled to a Majorana nanowire with MBSs prepared at its opposite ends. The system can be described by the following Hamiltonian [6,8,42,45,46],

(1) $$\begin{array}{ccc} H & & =\varepsilon_{d}d^{†}d+\sum_{k\beta \sigma }\varepsilon_{k\beta }C_{k\beta ,\sigma }^{†}C_{k\beta ,\sigma }+\sum_{k\beta }\Delta \left(C_{k\beta ,↓}C_{-k\beta ,↑}+H.c.\right)\\ & & +\sum_{k\beta \sigma }\left(V_{k\beta }e^{i\phi_{\beta }/2}C_{k\beta ,\sigma }d+H.c.\right)+H_{MBSs}, \end{array}$$

where the first term in the right of Equation (1) is for the QD with quantized energy level $\varepsilon_{d}$ and creation (annihilation) operator $d^{†}\left(d\right)$. The second and third terms are for the superconductors acting as leads with energy gap Δ, quasi-particle energy $\varepsilon_{k\beta \sigma }$ (β=L/R, spin σ=↑/↓, wave vector *k*) and creation (annihilation) operator $C_{k\beta \sigma }^{†}\left(C_{k\beta \sigma }\right)$ [6,8,47,48]. The forth term in the right side of Equation (1) stands for hopping between the QD and the superconductors with amplitude $V_{k\beta }$ and phase factor $\phi_{\beta }$. The last term in the right of Equation (1) is for the MBSs formed at the ends of the nanowire and their interaction with the QD [46,47,48,49,50],

(2) $$\begin{array}{c} H_{MBSs}=i\delta_{M}\eta_{1}\eta_{2}+\lambda \left(d-d^{†}\right)\eta_{1}, \end{array}$$

in which $\delta_{M}$ is the direct hybridization amplitude between the MBSs with operators $\eta_{1}$ and $\eta_{2}$. The quantity λ stands for hybridization strength between $\eta_{1}$ and the QD.

Following previous work [46], we make an unitary transformation to convert the MBSs into regular fermion representation by introducing $f=\left(\eta_{1}+i\eta_{2}\right)/\sqrt{2}$ and $f^{†}=\left(\eta_{1}-i\eta_{2}\right)/\sqrt{2}$. The Hamiltonian $H_{MBSs}$ then is rewritten as,

(3) $$\begin{array}{c} {\tilde{H}}_{MBSs}=\delta_{M}\left(f^{†}f+\frac{1}{2}\right)+\frac{\lambda }{\sqrt{2}}\left(d-d^{†}\right)\left(f+f^{†}\right). \end{array}$$

Since the dc Josephson current considered here arises from the differences in phase factors ($\phi =\phi_{L}-\phi_{R}$) and temperatures ($\Delta T=T_{L}-T_{R}$) between the left and right superconductors, the TPSC in the linear response regime then can be defined similarly to the charge Seebeck coefficient in the usual thermoelectric effect. To be specific, one firstly expands the Josephson current *J* with respective to infinitely small Δϕ and ΔT as

(4) $$\begin{array}{c} J=M\Delta \phi +K\Delta T, \end{array}$$

and then define the TPSC $S_{\phi }$ as the arisen phase difference Δϕ in response to the temperature difference, i.e., $S_{\phi }=-\Delta \phi /\Delta T$ under the condition of J=0. Therefore, we next calculate the Josephson current *J* by using the nonequilibrium Green’s function method. Following Refs. [14,15,51], the current can be expressed in terms of the Green’s function as,

(5) J=eh∫dεRe[Σ˜<Gda(ε)−Σ˜rGd<(ε)]11,

where $G_{d}^{a/<}\left(\varepsilon \right)$ the advanced/lesser Green’s function of the QD, and ${\tilde{\Sigma }}^{r/<}=\Sigma_{L}^{r/<}-\Sigma_{R}^{r/<}$ the difference between the retarded/lesser self-energies due to the left and right superconductors. We calculate the QD’s retarded/advanced Green’s function by adopting the Dyson equation method [6,15,16,52],

(6) $$\begin{array}{c} G_{d}^{r/a}\left(\varepsilon \right)={\left[\varepsilon 1_{2\times 2}-H_{dd}-\left(\Sigma_{L}^{r/a}+\Sigma_{R}^{r/a}\right)-\Sigma_{M}^{r/a}\right]}^{-1}, \end{array}$$

in which $1_{2\times 2}$ is an unitary matrix. The elements of the diagonal 2×2 matrix $H_{dd}$ are given by $H_{dd,11}=\varepsilon_{d}$ and $H_{dd,22}=-\varepsilon_{d}$. The retarded self-energy of the β-th superconductor is given by

(7) $$\begin{array}{c} \Sigma_{\beta }^{r}=-\frac{i}{2}\Gamma_{\beta }\rho \left(\varepsilon \right)\left(\begin{array}{cc} \;\;\;\;\;1 & -\frac{\Delta }{\varepsilon }e^{i\phi_{\beta }}\\ -\frac{\Delta }{\varepsilon }e^{-i\phi_{\beta }} & \;\;1 \end{array}\right), \end{array}$$

where $\Gamma_{\beta }=2\pi {\left|V_{k\beta }\right|}^{2}N_{\beta }$ is the hopping amplitude between the superconductors and the QD, with $N_{\beta }$ the density of states (DOS) in the normal state. The quantity ρ(ε) is the generalized DOS of the superconductors normalized by the normal state, and is defined as

(8) $$\begin{array}{c} \rho \left(\varepsilon \right)=\left\{\begin{array}{cc} \frac{\left|\varepsilon \right|}{\sqrt{\varepsilon^{2}-\Delta^{2}}} & \;\;\;\;\;|\varepsilon |>\Delta \\ -i\frac{\varepsilon }{\sqrt{\Delta^{2}-\varepsilon^{2}}} & \;\;\;\;\;|\varepsilon |<\Delta . \end{array}\right. \end{array}$$

The retarded/advanced self-energy $\Sigma_{M}^{r/a}$ in Equation (6) arises from the coupling between the MBSs and the QD, and is calculated in the matrix form as $\Sigma_{M}^{r/a}=H_{dM}g_{M}^{r/a}\left(\varepsilon \right)H_{Md}$ [52], in which

(9) $$\begin{array}{c} H_{dM}=\frac{\lambda }{\sqrt{2}}\left(\begin{array}{cc} \;\;-1 & -1\\ \;\;\;\;1 & \;\;\;1 \end{array}\right), \end{array}$$

and $H_{Md}=H_{dM}^{†}$. The matrix $g_{M}^{r/a}\left(\varepsilon \right)=\left(\varepsilon 1_{2\times 2}-H_{MM}\pm i0^{+}\right)$, and the elements of the diagonal matrix $H_{MM}$ are $H_{MM,11}=\delta_{M}$ and $H_{MM,22}=-\delta_{M}$. The lesser self-energy due to the superconductors are given by $\Sigma_{\beta }^{<}=f_{\beta }\left(\varepsilon \right)\left(\Sigma_{\beta }^{a}-\Sigma_{\beta }^{r}\right)$. The quantity $f_{\beta }\left(\varepsilon \right)=1/\left\{1+\exp\left[\left(\varepsilon -\mu_{\beta }\right)/k_{B}T_{\beta }\right]\right\}$ is the Fermi-Dirac distribution function with $\mu_{\beta }$ the Fermi energy in the β-th superconductor, Boltzmann constant $k_{B}$, temperature in the left/right superconductors $T_{L/R}=T\pm \Delta T/2$, in which *T* the system equilibrium temperature and ΔT the temperature difference that induces thermoelectric effect. After expanding *J* in Equation (5) with respective to Δϕ and ΔT to the first order and taking Equation (4) into consideration, one obtains [6,8],

(10a) $$\begin{array}{ccc} & & M=\frac{e}{h}\int d\varepsilon j_{c}\left(\varepsilon \right), \end{array}$$

(10b) $$\begin{array}{ccc} & & K=\frac{e}{h}\int d\varepsilon \left[j_{p}\left(\varepsilon \right)+j_{qp}\left(\varepsilon \right)\right], \end{array}$$

where the current carrying density of states (CCDOS) are individually given by [15,16]

(11a) jc(ε)=Γs2Δ2ε2−Δ2Im[1A(ε)]f(ε),

(11b) jp(ε)=ΓsRe[ρ(ε)]Im[−Gd,11r(ε)]dfdT,

(11c) jqp(ε)=(ΓsΔε)2Re[ρ(ε)]Re[−ρ(ε)A(ε)]cos2ϕ2dfdT,

where $A\left(\varepsilon \right)=\left[\varepsilon +\varepsilon_{d}-\left(\Sigma_{L,22}^{r}+\Sigma_{R,22}^{r}+\Sigma_{M,22}^{r}\right)\right]\left[\varepsilon -\varepsilon_{d}-\left(\Sigma_{L,11}^{r}+\Sigma_{R,11}^{r}+\Sigma_{M,11}^{r}\right)\right]-\left(\Sigma_{L,12}^{r}+\Sigma_{R,12}^{r}+\Sigma_{M,12}^{r}\right)\left(\Sigma_{L,21}^{r}+\Sigma_{R,21}^{r}+\Sigma_{M,21}^{r}\right)$, equilibrium Fermi-Dirac function $f\left(\varepsilon \right)=1/\{1+\exp\left[\left(\varepsilon -\mu \right)/k_{B}T\right]\}$ with $\mu_{L}=\mu_{R}=\mu$, and $\Gamma_{s}=\Gamma_{L}=\Gamma_{R}$. The TPSC then is calculated by $S_{\phi }=-K/M$. Finally in this section, we note that a Dynes Broadening is added in the superconducting gap energy to avoid divergence, i.e., $\Delta =\Delta_{0}-i\mathrm{sign}\left(\varepsilon \right)\eta$, and η is fixed to be $10^{-4}$ in the following numerical calculations [6].

## 3. Numerical Results

**Figure 2:**
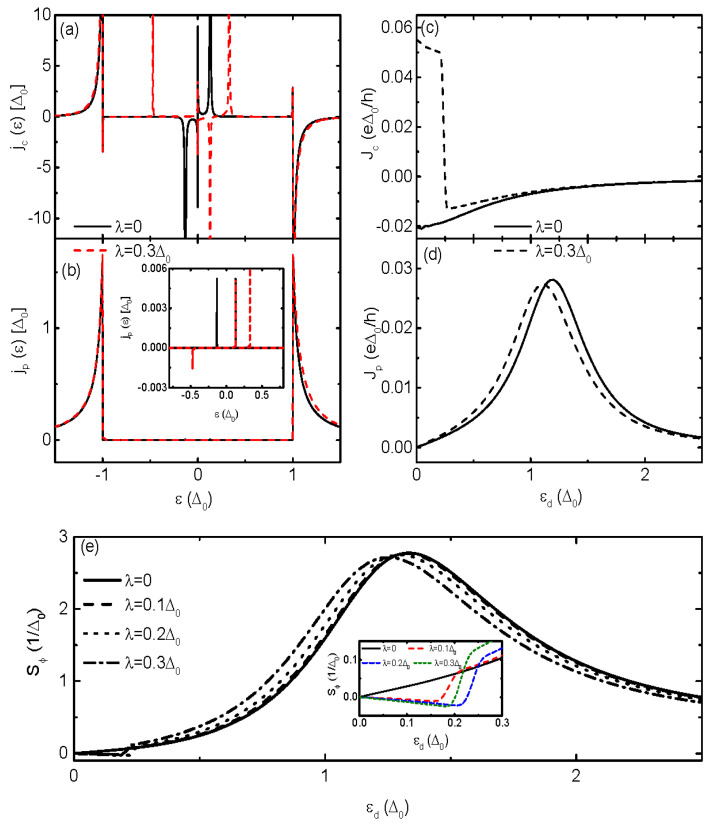
(Color online) The CCDOS $J_{c}\left(\varepsilon \right)$ in (**a**), $J_{p}\left(\varepsilon \right)$ with a inset of it in (**b**) as functions of the dot level for different values of λ. (**c**,**d**) are for the corresponding Josephson and quasi-particle currents, respectively. (**e**) is for the TPSc and its blowup for the chosen values of λ. Other parameters are $\Gamma_{s}=0.15\Delta_{0}$, equilibrium temperature $T=0.2\Delta_{0}$, and MBS-MBS coupling strength $\delta_{M}=0$.

In this section, we set $\Delta_{0}\equiv 1$ as the energy unit, and choose the Fermi level in the superconductors as zero (reference) energy (μ=0). We focus on the sign change of the TPSC induced by physical quantities related to the MBSs, such as QD-MBS coupling strength λ, direct overlap between the MBSs $\delta_{M}$, and the hooping between the QD and the superconductors $\Gamma_{s}$. To show the uniqueness of the sign reversion of the TPSC induced by the MBSs, we also examine the cases in structure of S-DQD-S. Figure 2a,b show individually the Josephson and quasi-particle CCDOSs in system of S-MQD-S for zero and finite λ. As is shown by the black solid curve in Figure 2a, there are two pairs of resonant peaks in $j_{c}\left(\varepsilon \right)$ within the superconducting gap. The peaks arise from the ABSs $E_{i}^{\pm }$ which are paired with energy of opposite signs and carry the discontinuous Josephson current in the form of $J_{dis}=\left(-2e/h\right)\sum_{i,\pm }f\left(E_{i}^{\pm }\right)\partial E_{i}^{\pm }/\partial \phi )$ [13,14,15,16]. Two broad peaks emerge around the states around ε=±Δ, which carry the continuous Josephson current. In fact, the peaks within the superconducting gap are much higher than that around ε=±Δ [6]. At zero temperature (T=0), the current is carried by ABSs of negative energy $E_{i}^{-}<=\mu$. For finite temperature, the current is contributed from ABSs located at negative energy to several $k_{B}T$. Correspondingly, the Josephson current $J_{c}<0$ as is indicated by the solid curve in Figure 2c. As for the quasi-particle CCDOS, its main contribution comes from states outside of the superconducting gap, where $j_{p}\left(\varepsilon \right)$ has two broad positive peaks which result in positive current $J_{p}$ as shown by the black solid line in Figure 2d. We emphasize that $j_{p}\left(\varepsilon \right)$, which is proportional to the real part of ρ(ε) as shown in Equation (11b), is mainly determined by energy states in the regimes of |ε|>Δ. But the ABSs within the superconducting gap, which are indicated by the inset in Figure 2b, also contribute to $J_{p}$ due to the Dynes broadening added to Δ [6]. When the QD is coupled to the MBSs (λ≠0), Figure 2a shows that the twin peaks in $j_{c}\left(\varepsilon \right)$ originally positioned individually at negative and positive energy regimes are shifted simultaneously to positive energy regime. Meanwhile, a sharp positive peak emerges below the Fermi level ε<μ as is shown by the red dash line in Figure 2a. The peaks around ε=±Δ are almost unchanged. Correspondingly, $J_{c}$ is positive for small value of the dot level $\varepsilon_{d}$, as is indicated by the dash curve in Figure 2c. As for $j_{p}\left(\varepsilon \right)$ in Figure 2b, the positive broad peaks outside the superconduting gap keep almost unchanged in the presence of λ. A new negative peak emerges within the superconducting gap with those two positive ones are changed in positions and height. Therefore, $J_{p}$ are suppressed slightly in magnitude as is shown by the dash curve in Figure 2d.

In Figure 2e we find that the magnitude of the TPSC $S_{\phi }$ is slightly changed by the coupling between the QD and the MBSs. It has a broad peak as the dot level $\varepsilon_{d}$ is at the tail of the superconducting gap singularity $\varepsilon =\Delta +\alpha \Gamma_{s}$ with the factor α∼ 1–2.5 [8]. The line-shape of the TPSC is very similar to that in systems composing of a QD sandwiched between normal metal leads [1,2]. The QD-MBSs coupling induces a weak sign change of TPSC due to the sharp peak in $j_{c}\left(\varepsilon \right)$ arisen from the presence of MBSs, which can be seen from the inset in Figure 2e. Here we note that in the case of λ=0, $j_{qp}\left(\varepsilon \right)$ is a odd function of ε due to the particle-hole symmetry, and has no contribution to the current [5,6]. In the presence of MBSs, the system’s particle-hole symmetry is broken and $J_{qp}$ is finite. But its value is negligibly small and then we did not show it. Moreover, both the currents and the TPSC are antisymmetrical with respective to $\varepsilon_{d}=0$, and thus we only show $S_{\phi }$ in half-interval of positive dot level $\varepsilon_{d}>0$ [6,8].

**Figure 3:**
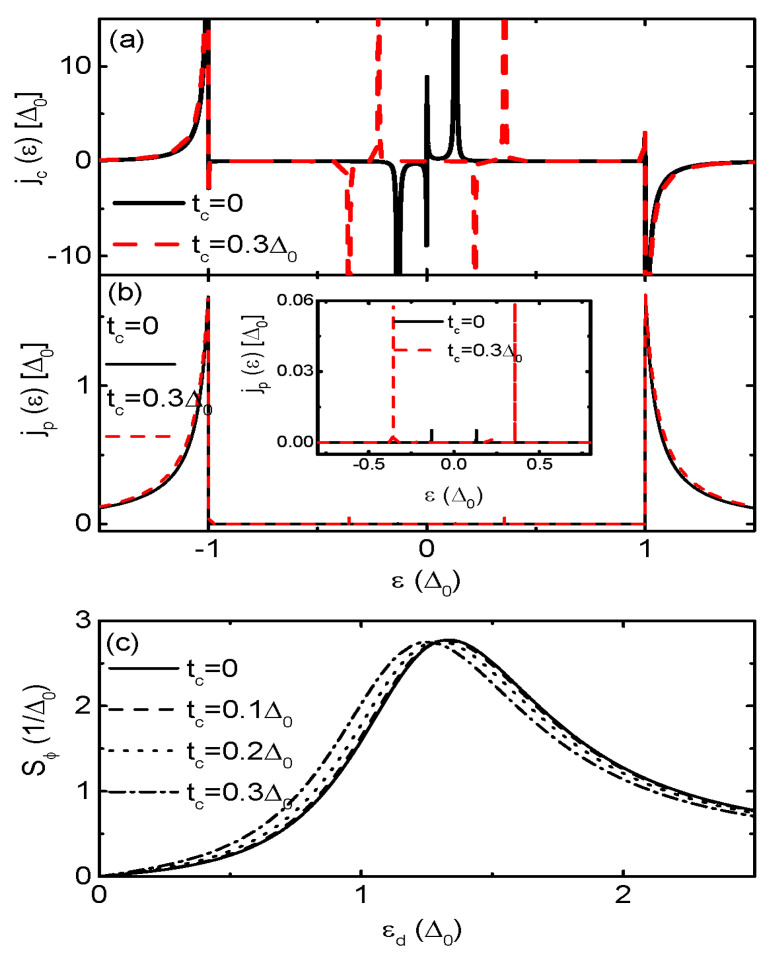
(Color online) The CCDOS i ndividually for $j_{c}\left(\varepsilon \right)$ in (**a**), $j_{p}\left(\varepsilon \right)$ in (**b**), and the TPSC in (**c**) as function of $\varepsilon_{d}$ for the case of S-DQD-S, i.e., the S-QD-S is coupled to another QD behaving as a regular fermion. The energy level of the side-coupled dot is denoted by $\delta_{M}$, which is set to be zero in this figure. Other parameters are as in Figure 2.

We now study in Figure 3 the system of S-DQD-S [8,16] in which the QD is coupled to regular fermion (another QD) in stead of the MBSs. To be convenient, we use the symbol $\delta_{M}$ to denote the energy level of the side-coupled QD, and the quantity $t_{c}$ represents for the inter-dot tunneling coupling strength. Detailed formulae for the Green’s function and the current can be found in previous work [8,16], and we here do not write them out for the sake of concision. Figure 3a,b indicate that the behaviors of $j_{c}\left(\varepsilon \right)$ and $j_{p}\left(\varepsilon \right)$ in structure of S-DQD-S are quite different from those in S-MQD-S shown in Figure 2a,b. When $t_{c}\neq 0$, the ABSs within the superconducting gap in $j_{c}\left(\varepsilon \right)$ are perfectly paired with energies of opposite signs, see the red dash curve in Figure 2a. Therefore, the Josephson current $J_{c}$ keeps almost unchanged by variation of the coupling between the QDs in the present case [8], which is not shown here. The CCDOS $j_{p}\left(\varepsilon \right)$ in Figure 3b changes indistinctively during the energy regime of |ε|>Δ, and the peak within the superconducting gap in the negative (positive) energy regime is shifted to deeper (higher) energy regime [8]. As a result of it, the broad resonance in $J_{p}$ is shifted slightly to the superconducting energy gap singularity $\varepsilon ∼\Delta_{0}$. The resonance in $S_{\phi }$ also moves toward this state with almost unchanged magnitude as is shown in Figure 3c. We emphasize that the sign of $S_{\phi }$ never changes regardless of the value of λ, which is different from the case in S-MQD-S.

**Figure 4:**
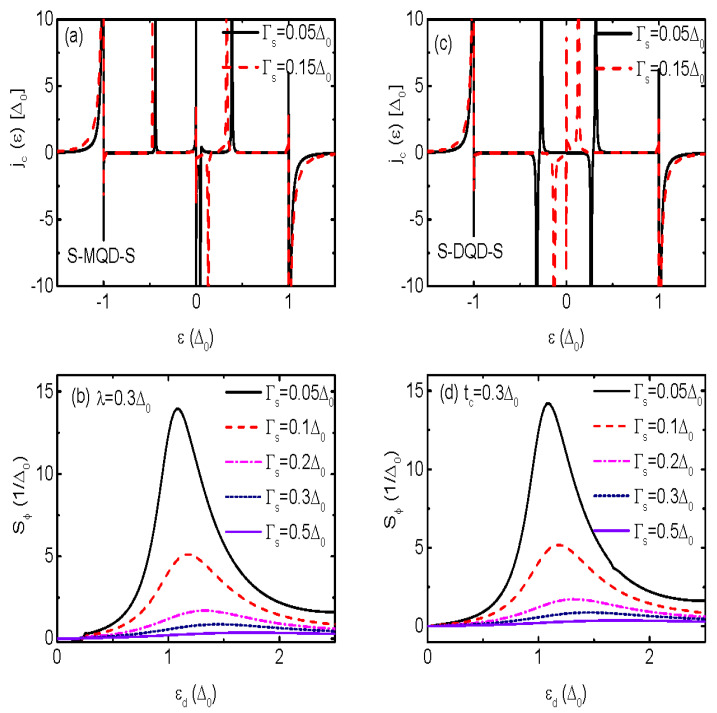
(Color online) The CC DOS $j_{c}\left(\varepsilon \right)$ in (**a**) *versus* electron energy and TPSC in (**b**) as a function of dot level for the system of S-MQD-S. (**c**,**d**) are individually $j_{c}\left(\varepsilon \right)$ and TPSC for the structure of S-DQD-S. The equilibrium temperature is $T=0.2\Delta_{0}$ and the MBS-MBS coupling strength $\delta_{M}$, which also denotes the energy level of the side-coupled dot, is set to be zero.

The coupling strength between the QD and the superconductors $\Gamma_{s}$ induces particle-hole splitting of the effective quasiparticle states formed at $\pm \sqrt{\varepsilon_{d}^{2}+\Gamma_{s}^{2}}$ [51]. With increasing value of $\Gamma_{s}$, the broad peaks in $j_{p}\left(\varepsilon \right)$ around $\varepsilon \approx \pm \Delta_{0}$ then are shifted individually to deeper and higher energy regimes, respectively. Therefore, the current’s contribution from the state around $\varepsilon \approx \Delta_{0}$ becomes weaker, resulting in reduced $J_{p}$. As for the CCDOS $j_{c}\left(\varepsilon \right)$, the splitting of the effective quasiparticle states has less impact on it. It is found that the peaks’ positions in $j_{c}\left(\varepsilon \right)$ for both S-MQD-M in Figure 4a and S-DQD-S in Figure 4b are shifted by $\Gamma_{s}$, but with negligibly small change in magnitude. We note that in the present case, the TPSC does not its sign in either S-MQD-S or S-DQD-S, which are shown in Figure 4b,d. The reason is that $\Gamma_{s}$ mainly induces splitting of the effective quasiparticle states and variation of the peaks’ position in $j_{c}\left(\varepsilon \right)$ [51]. Both of these two effects will not result in sign change of the current. Moreover, Figure 4b,d indicate that the magnitude and line-shape of $S_{\phi }$ in the two types of structures are almost the same regardless of the value of $\Gamma_{s}$.

**Figure 5:**
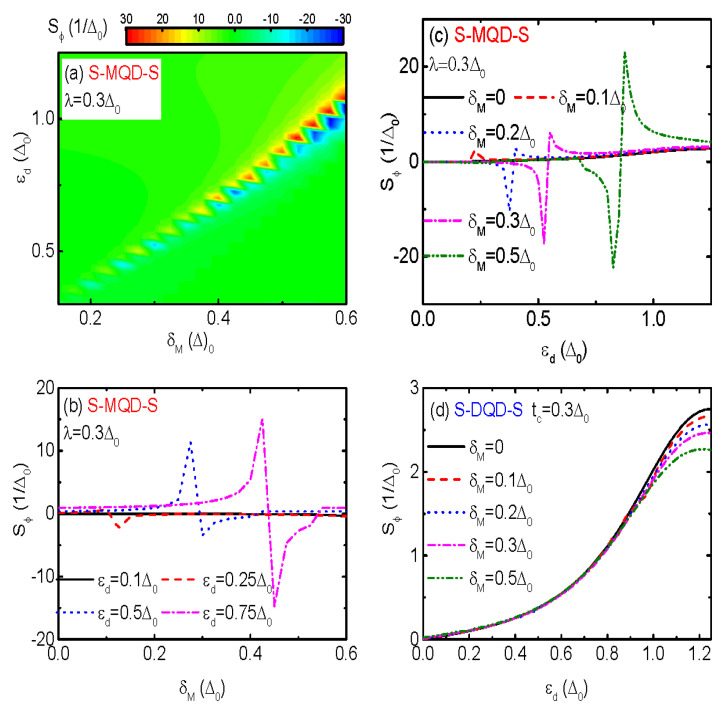
(Color online) Counter plot of the TPSC *versus* $\varepsilon_{d}$ and $\delta_{M}$ (**a**). (**b**,**c**) are the TPSC varying with $\delta_{M}$ and $\varepsilon_{d}$, respectively. For comparation, TPSC *versus* $\varepsilon_{d}$ is shown in (**d**) for different values of $\delta_{M}$. The equilibrium temperature in all the above figures are set to be $T=0.2\Delta_{0}$.

The change of $S_{\phi }$ from positive to negative value induced by λ, which may indicate the presence of MBSs, is rather weak in magnitude and occurs in a narrow dot level regime. This is then difficult to be detected in experiments. The two modes of MBSs overlaps to each other with an amplitude depending on the Majorana nanowire’s length, i.e., $\delta_{M}∼e^{-L_{M}/\xi }$ where $L_{M}$ denotes the length of the nanowire and ξ the superconducting coherence length. In previous work [39], López et al. found that $\delta_{M}$ in NM-MQD-NM will lead to sign reversion of the thermopower. The reason is that in the presence of dot-MBSs coupling (λ≠0), the Majorana level $\left(\delta_{M}\right)$ and dot level $\left(\varepsilon_{d}\right)$ repels each other. In the present S-MQD-S device, we find in Figure 5a that there are a series of zero points in the TPSC $\left(S_{\phi }=0\right)$, at whose two sides emerge sharp peaks with opposite signs. Moreover, the positions of the zero points as well as the peaks are almost linear with either dot level $\varepsilon_{d}$ or Majorana level $\delta_{M}$, which can be seen more clearly in Figure 5b,c. As is shown in Figure 5b, the zero point in $S_{\phi }$ emerges given that $\varepsilon_{d}\approx \lambda +\delta_{M}$, and two sharp peaks with opposite signs emerge at the two sides of each zero point. As is compared to the case of $\delta_{M}=0$, the peak’s height is also significantly enhanced. The relation between $\varepsilon_{d}$ and $\delta_{M}$ is confirmed by the results shown in Figure 5c, in which the zero points in $S_{\phi }$ emerge at about $\delta_{M}\approx \varepsilon_{d}-\lambda$.

We note that the sign change of $S_{\phi }$ in the present paper is quite different from that of the thermopower *S* in NM-MQD-NM [1,2]. Firstly, the sign change in $S_{\phi }$ occurs under the condition of $\delta_{M}\geq k_{B}T$ as is shown in Figure 5a–c; the sign change in *S*, however, appears in the case of $\delta_{M}\leq k_{B}T$ [1,2]. Secondly, $S_{\phi }$ changes its sign at a series of $\varepsilon_{d}$, whose value depends on $\delta_{M}$; but *S* changes its sign only at $\varepsilon_{d}=0$. Thirdly, $S_{\phi }$ develops sharp high peaks at the two sides of the zero points, whereas *S* changes rather smoothly around the zero point. We attribute the exotic behavior of $S_{\phi }$ to the newly developed ABSs states within the superconducting gap induced by the MBSs, a mechanism that is quite different from that in system of NM-MQD-NM [1,2]. For comparison, we present the TPSC $S_{\phi }$ in S-DQD-S varying with respective to the dot level for different values of the energy levels of the side-coupled dot $\delta_{M}$ in Figure 5d. It shows that $S_{\phi }$ depends weakly on $\delta_{M}$ when the central dot’s energy level is within the superconducting gap ($\varepsilon_{d}\leq \Delta_{0}$), with unchanged sign regardless of the value of $\delta_{M}$ [8]. Around the tail of the sperconducting gap $\Delta_{0}$, the magnitude of the resonance in $S_{\phi }$ is monotonously reduced by increasing $\delta_{M}$.

**Figure 6:**
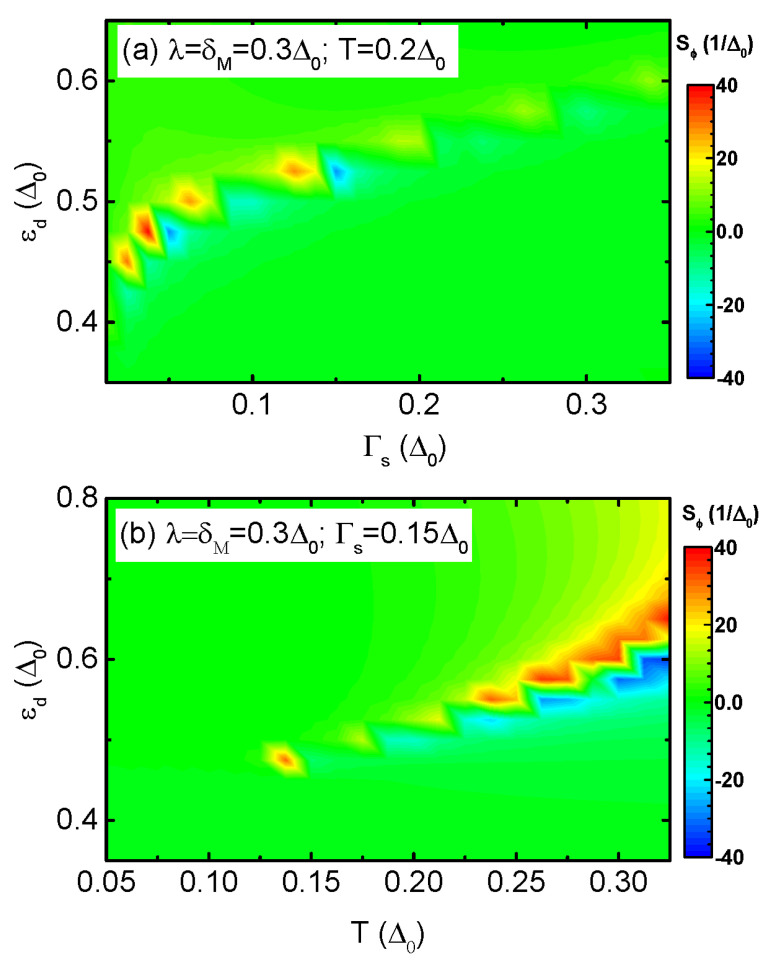
(Color online) TPSC varying with $\varepsilon_{d}$ and $\Gamma_{s}$ in (**a**), and *versus* $\varepsilon_{d}$ and *T* in (**b**), respectively. From (**a**,**b**), one can see that $\Gamma_{s}$ and *T* may induce sign change of TPSC in S-MQD-S. In S-DQD-S, however, they can not change the sign of TPSC, which are then not shown.

We revisit the impacts of $\Gamma_{s}$ on $S_{\phi }$ for finite value of $\delta_{M}$ in Figure 6a. Different from the case of $\delta_{M}=0$ in Figure 4b, we find that when the MBSs are overlapped with each other $\left(\delta_{M}\neq 0\right)$, the sign of $S_{\phi }$ is reversible by $\Gamma_{s}$. Similar to the case in Figure 5a, zero points emerge in $S_{\phi }$ and a pair of sharp peaks with opposite signs are induced. The sign change of $S_{\phi }$ occurs under the conditions of $\Gamma_{s}\leq k_{B}T$ and $|\varepsilon_{d}|<\Delta_{0}$. We attribute this phenomenon to the subtle splitting and shifting of the ABSs within the superconducting gap due to the hybridization between the QD and the MBSs. It is known that the variation of equilibrium temperature *T* will result in sign change of the thermopower in the usual thermoelectric effect [1,2]. In Ref. [6], it was also shown that the sign of $S_{\phi }$ depends on *T*. Figure 6b shows that both the sign and magnitude of $S_{\phi }$ strongly depends on the value of *T* and $\varepsilon_{d}$ in the present S-MQD-S system. Moreover, the sign change of $S_{\phi }$ occurs when $k_{B}T\geq \Gamma_{s}$, which is in consistent with the result shown in Figure 6a. The above results may arise from the fact that different channels participate in transport process and manifest their functions depending on the value of system temperature, dot level, as well as the coupling strength between the QD and the superconductors mediated by the MBSs.

## 4. Summary

In summary, we have studied thermoelectric effect in a Josephson junction with a QD sandwiched between the left and right superconductor leads and side-coupled to a nanowire with MBSs prepared at its ends. We focus our attention on the phase difference between the two superconductors arisen from the thermal bias applied across them, which is the TPSC. Our numerical results show that both the sign and magnitude of TPSC are sensitive to the possible existence of MBSs, and thus provides a new way to detect the MBSs with half-fermionic MBSs nature. The sign change of the TPSC is attributed to the newly developed ABSs due to the coupling between the QD and MBSs. The positions and peaks’ height of these ABSs can be adjusted by the direct hybridization between the MBSs, coupling between the QD and the superconductors, as well as the system equilibrium temperature, and thus alter the sign and magnitude of the TPSC. If the half-fermionic MBSs side-coupled to the QD is replaced by regular fermions, i.e., another QD, there is no sign change in the TPSC. Moreover, the magnitude of the TPSC also changes rather smoothly by the variation of system parameters in the S-DQD-S.

## Data Availability

All data included in this study are available upon request by contact with the corresponding author.
